# Observing pedestrian-vehicle traffic conflicts in school zones to evaluate the effectiveness of road safety interventions and reduce injuries in Ghana, Vietnam, and Mexico, 2019-2021

**DOI:** 10.5249/jivr.v14i3.1710

**Published:** 2022-07

**Authors:** Jennifer M. Swanson, Natalie Draisin, Agnieszka Krasnolucka, Clara Vadillo, Sonia Medina, Berenice Pérez, Simon Kalolo, Bui Nguyen Thu Quyen, Vo Ngoc Minh, Erin Sauber-Schatz

**Affiliations:** ^*a*^ Centers for Disease Control and Prevention, Atlanta, GA, United States.; ^*b*^ FIA Foundation, London, United Kingdom.; ^*c*^Institute for Transportation and Development Policy, Mexico City, Mexico.; ^*d*^ Amend, Accra, Ghana.; ^*e*^ Asia Injury Prevention Foundation, Ho Chi Minh City, Vietnam.

**Keywords:** Traffic conflicts, School zones, Low- and middle income countries, Evaluation, Student pedestrians

## Abstract

**Background:**

Daily more than 3,000 children are injured or killed on the road, often along the school route. Road traffic crashes and resulting injuries are preventable. More can be done to reduce injuries and save lives. Traffic Conflict Techniques (TCTs) are simple methods of collecting observational data to evaluate the effectiveness of road safety interventions through counting and analyzing traffic conflicts. A TCT Toolkit was developed and piloted to analyze pedestrian-vehicle traffic conflicts in school zones in low- and middle-income countries.

**Methods:**

Three non-governmental organizations in Ghana, Vietnam, and Mexico applied three TCTs from the TCT Toolkit to collect traffic conflict data before (pre-intervention) and after (post-intervention) road safety intervention implementation. As the number of traffic conflicts was often less than 100, confidence intervals (CIs) based on gamma distributions were calculated for the traffic conflict rate. Using the calculated traffic conflict rate, the difference between pre- and post-intervention rates was assessed by determining overlap of the CIs. When CIs did not overlap, the difference was said to be statistically significant at the 0.05 level.

**Results:**

For each method, results indicated a decrease in traffic conflicts between pre- and post-intervention data collection periods. Pre- and post-intervention traffic conflict rates with non-overlapping CIs demonstrated the results were statistically significant, providing evidence that the road safety interventions were effective.

**Conclusions:**

TCTs are relatively low-cost and simple techniques that provide an opportunity to base road safety improvement decisions on real-world data. TCTs are effective in objectively evaluating road safety interventions and can help decision-makers evaluate strategies for improving road safety, preventing injuries and saving lives.

## Introduction

Annually, road traffic crashes are responsible for more than 1.35 million deaths globally and over half (54%) of deaths are among vulnerable road users.^1^ Beyond deaths, an additional 20-50 million people are nonfatally injured in crashes each year.^2^ In 2016, 93% of road traffic deaths occurred in low- and middle-income countries (LMICs), despite these countries having only 60% of the world’s vehicle fleet.1 Globally, road traffic injuries are the leading cause of death among people aged 5 to 29 years.^1^

Daily over 3,000 children are injured or killed on roads, often along the school route.^3^ Children encounter unsafe roads within school zones increasing their risk of being in a crash, injured or killed. Police records and crash surveillance systems are retroactive approaches used to identify unsafe roads or intersections and make decisions on risk reduction strategies. A timelier proactive approach is to collect and analyze traffic conflict data. A traffic conflict occurs when two or more road users are on a collision course and risk crashing if one of the road users does not take evasive action (e.g., swerve) to change their movement/trajectory thus avoiding a crash.^4,5^ Traffic conflict data can provide objective evidence about suitable road safety intervention(s) and establish a baseline for evaluation to determine effectiveness of the intervention(s). Traffic Conflict Techniques (TCTs) are simple methods to help decision makers select and evaluate effective strategies for improving road safety and preventing crashes. 

This paper focuses on pedestrian-vehicle traffic conflicts between non-motorized student road users (e.g., pedestrians, cyclists) and drivers of motorized vehicles (e.g., cars, buses, motorized 2- and 3-wheelers) occurring in and around primary and secondary school zones in LMICs. We describe three methods of collecting pedestrian-vehicle traffic conflict data and provide case examples of how local non-governmental organizations (NGOs) have applied the methods to improve road safety in school zones in Ghana, Vietnam, and Mexico. 

## Methods 

We utilized the “Traffic Conflict Technique (TCT) Toolkit: Making the Journey to and from School Safer for Students”.^1^ This TCT Toolkit is intended to serve as a comprehensive guide for applying TCTs and provides five methods to evaluate the impact of road safety interventions. This paper focuses on three of the more complex methods in the toolkit. A summary of each method is below; more detailed descriptions are in the toolkit.

***Traffic Conflict Technique Methods***

**Method 1:**

Version 1: Institute of Highways and Transportation Conflicts Technique (IHTCT) Pedestrian-Vehicle Conflict Technique categorizes the severity of a traffic conflict based on four classifications, ranked 1-4, within four factors: Factor A (Time to Collision), Factor B (Severity of Evasive Action), Factor C (Complexity of Evasive Action), Factor D (Distance to Collision).^6^ Once a classification is assigned (A-D), these four vertically ordered numbers provide a final sequence. This sequence is used to determine the conflict severity, ranging between Grade 1 (slight) to Grades 2-4 (serious). 

**Method 2:**

Version 2: Institute of Highways and Transportation Conflicts Technique (IHTCT) Pedestrian-Vehicle Conflict Technique first records each pedestrian-vehicle conflict as one of two types of interactions pertaining to the speed of the vehicle: 1) Steady Care-Pedestrian (SC-P) – the vehicle is traveling at a steady speed at the time of interaction with the pedestrian, and 2) Effective Shared Space (ESS) – the vehicle is stopped or travelling at very slow speeds.^7^ A severity grading for both the pedestrian and vehicle is then recorded using three criteria: change in speed, change in direction, and vehicle acceleration.

**Method 3:**

The Swedish Traffic Conflict Technique (TCT) requires two measurements-speed and distance-to determine the severity of a pedestrian-vehicle conflict.^8^ The best way to measure pedestrian and vehicle speed and distance is through video recording or in-field observation. Experts of the Swedish TCT believe judging vehicle speed is relatively reliable with skill honed by practice. The speed of the road user is estimated at the moment the first road user (RU1) takes evasive action (swerve, slow down), with distance estimated from the exact location where RU1 takes the evasive action to where the collision would have occurred if the evasive action had not been taken. The data collector estimates the speed at the exact moment RU1 avoids collision and at the same time estimates the distance from RU1 to the point where a collision would have occurred without evasive action. Speed and distance are used to identify a Time-to-Accident (TA) indicator (time remaining for RU1 to successfully perform an evasive action), which is graphed with the speed on a severity curve to determine if the conflict is serious or non-serious. 

***Data collection: road user and traffic conflict counts***

In Ghana, Vietnam, and Mexico, road user counts and traffic conflict counts were collected at each school location on a typical day (non-holiday) to assess traffic conflict burden and change after road safety intervention implementation. Data collection occurred at three points throughout the study. First, road user counts were collected. Data collectors counted the number of pedestrians and vehicles passing through the data collection site, generally occurring one week before and during the same time as planned pre-intervention traffic conflict data collection. These counts provided baseline road user counts and served as a reference when analyzing and interpreting data. The analyst chose one road user (e.g., drivers) to use throughout analysis. Second, pre-intervention traffic conflict data were collected using method-specific data collection forms. A video camera was used to provide opportunity to review conflicts to ensure data quality.^2^ Third, post-intervention traffic conflict data were collected; ideally occurring at multiple time points (at least one-month, but ideally 3-months and 6-months post-intervention). 

***Study Sites***

Detailed data collection and infrastructure information is provided in Table 1. Each NGO applied a TCT method at a designated location within a school zone. Amend^3^ selected the Oblogo Cluster of Schools located in Accra, Ghana; the Asia Injury Prevention (AIP) Foundation^4^ selected Minh Duc School in Ho Chi Minh City, Vietnam; and the Institute for Transportation and Development Policy (ITDP)^5^ selected the intersection of Boulevard Hermanos Serdán and Street Nte 37 within the school zone of the Benemérito Normal State Institute “Gral. Juan Crisóstomo Bonilla” (BINE) in Puebla de Zaragoza, Puebla, Mexico. 

**Table 1:** Explanation of data collection and infrastructure modifications; Ghana, Vietnam, Mexico, October 2019-February 2021.

	Non-Governmental Organization		
	Amend (Ghana)	AIP Foundation (Vietnam)	ITDP (Mexico)
TCT Method	Version 1: IHTCT	Version 2: IHTCT	Swedish TCT
**Vehicle counts **			
Month	January 2020	October 2019	May 2019
Day(s)	Thursday	Friday	Tuesday
Time	6:30-7:30am & 2:00-3:00pm	6:10-7:00am & 4:15-5:00pm	1:00-2:00pm
**Pre-intervention**			
Month	January 2020	October 2019	October 2019
Day(s)	Tuesday & Wednesday	Friday & Monday	Monday & Tuesday
Time	6:30-7:30am & 2:00-3:00pm	6:10-7:00am & 4:15-5:00pm	6:30-7:30am & 1:00-2:00pm
**Intervention Implementation**			
Month	March 2020	June-July 2020	November 2019
Infrastructure	1) Installation of: speed humps, road signs, painted pedestrian crossings and walkways, crash barriers	1) Installation of: “School Zone Ahead” sign, two solar flashing beacons	1) Sidewalk extensions
changes	2) Reduced speed limit from 50 to 30km/hr	2) Construction of two refuge islands at marked crosswalks	2) Crosswalk markings
	3) Provided site-specific road safety education	3) Painted traffic calming and “Slow Down” markings	3) Implementation of bollards to separate pedestrian/vehicle areas
**Post-intervention**			
Month	February 2021	October 2020	January 2020
Day(s)	Tuesday & Wednesday	Thursday	Tuesday^8^ & Wednesday
Time	6:30-7:30am & 1:00-2:00pm^9^	6:15-7:00am & 4:15-5:00pm	6:30-7:30am & 1:00-2:00pm

Data collection occurred during the specific site’s morning and afternoon peak school travel times. For the intervention, the NGOs implemented various infrastructure changes. Amend implemented infrastructure changes because of results from pre-intervention data, community member consultations and technical advice from local authorities. AIP Foundation and ITDP previously planned infrastructure changes before traffic conflict study commencement.

***Data analysis***

Pre-intervention data analysis was conducted to help inform or validate selection of infrastructure interventions. Post-intervention data analysis was conducted and compared with pre-intervention results to determine effectiveness of the road safety interventions. 

The traffic conflict rate was calculated using the number of traffic conflicts divided by the number of hours the conflicts were counted (e.g., 4- or 2-hour period) and by the number of vehicle road users passing through the observation area (x 1,000). Traffic conflict counts may vary between measurement periods even when the true underlying traffic conflict rate is unchanged; that is, the counts are subject to random variation and this variability should be taken into account when assessing significance of change. 

For this study, the random variability is incorporated into the calculation of 95% confidence intervals (CIs) for traffic conflict rates. To simplify the CI calculations, the denominator of the rate (number of vehicles passing through the observation area) was assumed a constant (i.e., possessing negligible random variation). As the number of traffic conflicts was often <100, CIs based on gamma distributions were calculated for traffic conflict rate.^6^ The difference between the pre- and post-intervention rate was assessed by comparing the CIs. If the CIs do not overlap, the difference can be said to be statistically significant at the 0.05 level. Therefore, it can be concluded that the difference between the traffic conflict rates for pre- and post-intervention data is statistically significant.^7^

1. Funding to develop the TCT Toolkit was provided by FIA Foundation via the CDC Foundation. 

2. Videos were only used for the purpose of the study, securely stored and destroyed at the end of data validation.

3. Amend is a road safety organization with offices in Ghana, Mozambique and Tanzania dedicated to improving environments to deliver safe and healthy journeys.

4. The AIP Foundation is a nonprofit based in Vietnam dedicated to decreasing road crash causalities by providing safe interventions in LMICs.

5. ITDP is a global organization making cities more livable by designing and implementing transport systems and policy solutions.

6. The gamma distribution formula can be found in the National Vital Statistics Reports, Vol. 68, No. 9, June 24, 2019. 

7. Comparing CIs is a conservative method for statistical significance-the difference between two rates may be statistically significant even if CIs overlap. Take caution when interpreting no significant difference between two rates, especially when the lower and upper limits overlap only slightly.

8. Due to rain, ITDP collected data on Tuesday instead of Mon-day as planned.

9. The children left school earlier because of shortened school hours during the post-intervention period.

10. In Vietnam, post-intervention traffic conflict data collection occurred in one day over a 1.5-hour period. The assumption was 45-120 minutes of observation. 

## Results

***Method 1: Version 1: IHTCT Pedestrian-Vehicle Conflict Technique***

In Ghana, there was an average of 929 vehicles per hour through the study area. Pre-intervention observations resulted in 80 total conflicts per 4 hours (average 20 conflicts per hour) (Table 2). The pre-intervention traffic conflict rate was 21.5 conflicts per hour per 1,000 vehicles (95% CI: 17.1, 26.8) (Table 2). 

**Table 2:** Pre- and post-intervention results; Ghana, Vietnam, Mexico.

	Method 1 (Ghana)		Method 2 (Vietnam)		Method 3 (Mexico)	
	Pre-intervention	Post-intervention	Pre-intervention	Post-intervention	Pre-intervention	Post-intervention
Road User Counts	929 vehicles (average per peak hour)		6,202 vehicles (average per peak hour)		1,149 vehicles (afternoon peak hour)	
**# traffic conflicts (4-hour period) **	80	62	390	96^10^	147	35
**Traffic conflict rate (per hour per 1,000 vehicles)**	21.5	16.7	15.7	7.7	32.0	7.6
**95% CI**	(17.1, 26.8)	(12.8, 21.4)	(14.2, 17.4)	(6.3, 9.5)	(27.0, 37.6)	(5.3, 10.6)

In Ghana, the pre-intervention conflicts varied in severity. Based on the classifications, 18 conflicts were slight (Grade 1), and 62 conflicts were serious (52 Grade 2, 10 Grade 3) (Table 3).

**Table 3:** Version 1: IHTCT Pedestrian-Vehicle Conflict Technique pre-intervention results, Ghana, January 2020.

DATE	Time of Day	Severity Grade 1	Severity Grade 2	Severity Grade 3	Severity Grade 4	Total Conflicts
28-JAN-20	AM	10	14	2	0	26
	PM	3	11	5	0	19
29-JAN-20	AM	3	18	3	0	24
	PM	2	9	0	0	11
**TOTAL**		18	52	10	0	80

Post-intervention observations resulted in 62 total conflicts per 4 hours (average 15.5 conflicts per hour) (Table 2). The post-intervention traffic conflict rate was 16.7 conflicts per hour per 1,000 vehicles (95% CI: 12.8, 21.4) (Table 2). Post-intervention conflicts also varied in severity resulting in 29, 21, 9, and 3, Grade 1-4, respectively (Table 4). Grade 4 conflicts were not observed during the pre-intervention period, which could indicate that although traffic conflicts decreased from pre- to post-intervention, overall severity might have increased.

**Table 4:** Version 1: IHTCT Pedestrian-Vehicle Conflict Technique post-intervention results, Ghana, February 2021.

DATE	Time of Day	Severity Grade 1	Severity Grade 2	Severity Grade 3	Severity Grade 4	Total Conflicts
**9-FEB-21**	AM	11	8	3	0	22
	PM	5	4	1	1	11
**10-FEB-21**	AM	10	4	3	1	18
	PM	3	5	2	1	11
**TOTAL**		29	21	9	3	62

***Method 2: Version 2: IHTCT Pedestrian-Vehicle Conflict Technique***

In Vietnam, there was an average of 6,202 vehicles per hour through the study area. Pre-intervention observations resulted in 390 total conflicts (153 vehicle conflicts + 212 SC-P pedestrian conflicts + 25 ESS pedestrian conflicts) per 4 hours (average 97.5 conflicts per hour) (Table 5). Over half (51.9%) of the pedestrians changed speed and gave way to avoid a conflict. The pre-intervention traffic conflict rate was 15.7 conflicts per hour per 1,000 vehicles (95% CI: 14.2, 17.4) (Table 2).

**Table 5:** Version 2: IHTCT Pedestrian-Vehicle Conflict Technique pre-intervention results, Vietnam, October 2019 (per 4 hours).

	Criteria	Grade	SC-P Interactions	ESS Interactions
**Vehicle**	I(change in speed)	1 – full speed	15	-
		2 – slowed down	65	-
		3 – stop	39	-
	II (change in direction)	1 – continues along intended path	2	-
		2 – deviated	32	-
	III (vehicle acceleration)	1 – accelerate immediately	-	-
		2 – wait to clear	-	-
		3 – no change	-	-
**VEHICLE TOTAL**			**153**	**-**
**Pedestrian**	I (change in speed)	1 – continues at same speed	59	4
		2 – accelerate	36	-
		3 – give way	101	9
		4 – return	-	-
	II (change in direction)	1 – continues along intended path	-	1
		2 – deviated	16	11
		3 – return	-	-
**PEDESTRIAN TOTAL**			**212**	**25**

Post-intervention observations resulted in 96 total conflicts (39 vehicle conflicts + 53 SC-P pedestrian conflicts + 4 ESS pedestrian conflicts) per 2 hours (average 48 conflicts per hour) (Table 6). The post-intervention traffic conflict rate was 7.7 conflicts per hour per 1,000 vehicles (95% CI: 6.3, 9.5) (Table 2).

**Table 6:** Version 2: IHTCT pedestrian-vehicle conflict technique post-intervention results, Vietnam, October 2020 (per 1.5 hours).

	Criteria	Grade	SC-P Interactions	ESS Interactions
**Vehicle**	I(change in speed)	1 – full speed	9	-
		2 – slowed down	4	-
		3 – stop	19	-
	II (change in direction)	1 – continues along intended path	-	-
		2 – deviated	6	-
	III (vehicle acceleration)	1 – accelerate immediately	1	-
		2 – wait to clear	-	-
		3 – no change	-	-
**VEHICLE TOTAL**			**39**	**-**
**Pedestrian**	I (change in speed)	1 – continues at same speed	36	3
		2 – accelerate	1	-
		3 – give way	15	-
		4 – return	-	-
	II (change in direction)	1 – continues along intended path	-	-
		2 – deviated	1	-
		3 – return	-	-
**PEDESTRIAN TOTAL**			**53**	**4**

***Method 3: Swedish TCT***

**Figure 1:**
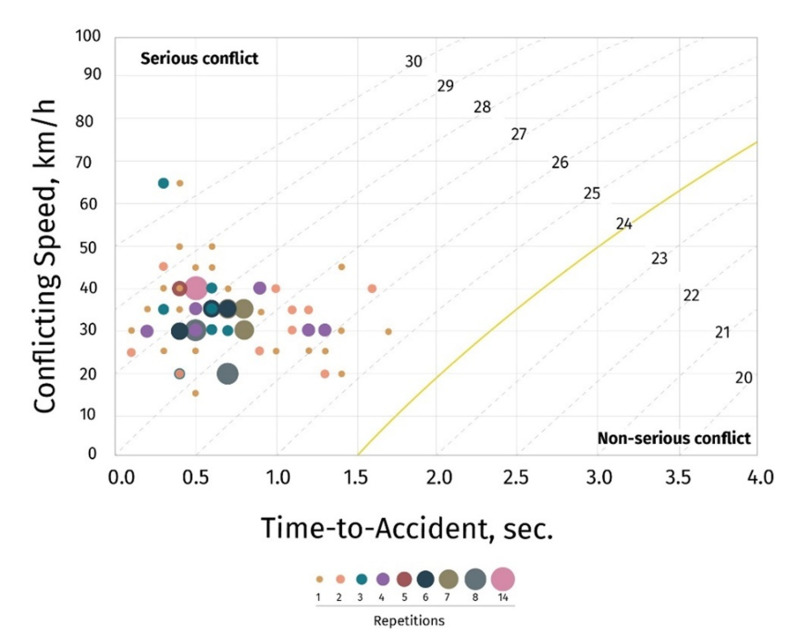
Swedish Traffic Conflict Technique pre-intervention conflict severity graph, Mexico, October 2019.

In Mexico, there was a total of 1,149 vehicles per hour through the study area. Pre-intervention observations resulted in 147 total conflicts per 4 hours (average 36.8 conflicts per hour) (Table 2). The pre-intervention traffic conflict rate was 32.0 conflicts per hour per 1,000 vehicles (95% CI: 27.0, 37.6) (Table 2). All 147 conflicts were considered serious as identified by the TA indicator with a severity score of >24 (Figure 1).

**Figure 2:**
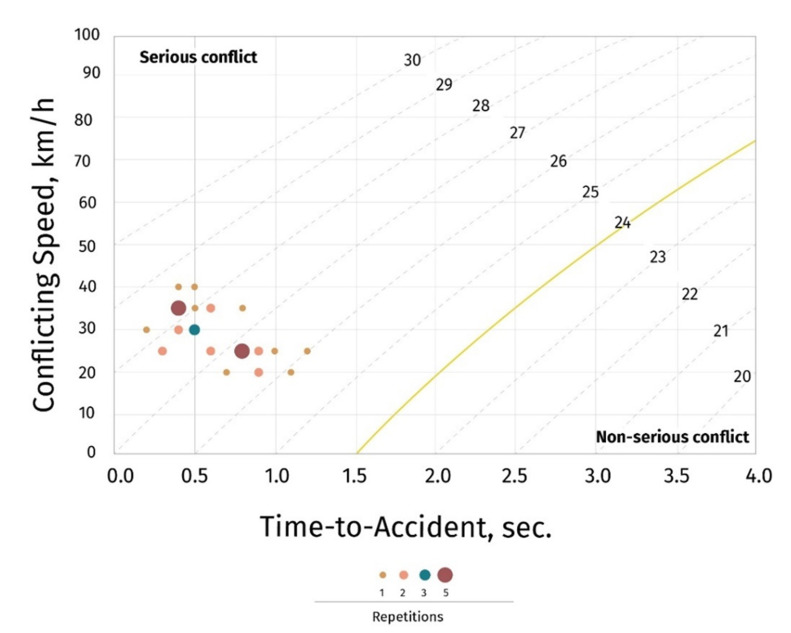
Swedish Traffic Conflict Technique post-intervention conflict severity graph, Mexico, January 2020.

Post-intervention observations resulted in 35 total conflicts per 4 hours (average 8.8 conflicts per hour) (Table 2). The post-intervention traffic conflict rate was 7.6 conflicts per hour per 1,000 vehicles (95% CI: 5.3, 10.6) (Table 2). Although the results indicate a decrease in total number of traffic conflicts between pre- and post-intervention, all 35 post-intervention conflicts were still considered serious (Figure 2).

## Discussion

Road traffic crashes and resulting injuries are preventable. In this paper, we presented a new evidence-based road safety resource and its application in three LMICs to make the journey to school safer. TCTs are methods of quickly collecting observational data to evaluate the effectiveness of road safety interventions by using real-world data and assessing the impact between pre- and post-intervention. 

Based on case studies in Ghana, Vietnam, and Mexico, TCTs achieved a timely evaluation of the impact of infrastructure interventions implemented in school zones. The results indicate these evaluations can be used in various geographical, cultural, and road safety settings. Since using TCTs is relatively simple, a rigorous evaluation will be more robust, but these observational data proved useful in proactively reducing traffic conflicts in these settings. Even in their simplest form, TCTs provide a way of basing road safety improvement decisions on real-world data and assessing the impact. Thus, TCTs can help decision-makers select and evaluate road safety interventions.

Due to the COVID-19 pandemic, more time passed between pre- and post-intervention data collection than previously planned in Ghana. Changes in traffic patterns and vehicle flow related to COVID-19 and infrastructure modifications might have affected post-intervention data, but this was not able to be assessed. 

Although TCTs are relatively simple, researchers must be properly trained on each method’s principles and theories. Without time dedicated to training, disparate interpretations are possible. In addition, it can be difficult to achieve data collector consistency and objectivity when estimating the conflict severity because of differences in personal perceptions. The severities also might have changed due to unforeseen variations in traffic patterns during the COVID-19 pandemic.

In Vietnam, inconsistency between pre- and post-intervention periods resulted in two days pre-intervention versus one day post-intervention-potentially altering the number of conflicts. One day of data collection could have artificially decreased the observed conflicts.

**Acknowledgements**

We acknowledge the collaborative contributions of Natalie Draisin and Agnieszka Krasnolucka at FIA Foundation. Several partners contributed to this paper: Clara Vadillo, Berenice Pérez, Sonia Medina, Gonzalo Peón Carballo with ITDP in Mexico City, Mexico; Simon Kalolo, Amma Oduro-Dankwah, Juliet Ado, Jeffrey Witte with Amend in Accra, Ghana; Minh Vo, Le Nguyen, Hong Bui, Quyen Bui, My Dang, Jimmy Tang, Mirjam Sidik with AIP Foundation in Ho Chi Minh City, Vietnam. Additionally, we thank Rose Rudd (CDC) for her analytic expertise.
